# Supplementary material for: Gastric intestinal metaplasia subtypes and the effects of c-Myc expression on severity
Source: PeerJ. 2025 Oct 31;13:e20257. doi: 10.7717/peerj.20257 (PMC12581917; doi:10.7717/peerj.20257)
Supplement: Supplemental Information 2 [file peerj-13-20257-s002.docx]

| 序号 | Serial Number | |
| --- | --- | --- |
| 病理号 | Pathological specimen number | |
| 姓名 | Name | |
| 性别（1男；2女） | Gender (1: male; 2 :female) | |
| 年龄 | Age(year oid) | |
| 年龄分层（1：＜40岁；2:40-60岁；3：≥60岁） | Age Stratification (1: < 40 years; 2: 40-60 years; 3: ≥ 60 years) | |
| 肠化分级（1轻；2中；3重） | Grading of the Severity of GIM (1:mild; 2:moderate; 3:severe) | |
| 肠化分型 | Types of GIM(1:complete GIM; 2:incomplete small intestinal metaplasia; 3:incomplete colonic metaplasia)) | |
| AB（0-；1+；2++；3+++） | AB staining（0-；1+；2++；3+++） | |
| PAS（0-；1+；2++；3+++） | AB–PAS staining（0-；1+；2++；3+++） | |
| HID（0-；1+；2++；3+++） | AB–HID staining（0-；1+；2++；3+++） | |
| C-myc阳性强度（0-；1+；2++；3+++） | Expression intensity of c-Myc cells(0:Negative; 1:Weakly Positive; 2: Positive; 3: Strongly Positive) | |
| C-myc百分比（%） | Percentage of c-Myc-positive cells(%) | |
| 低（0-10%）、中（10-30%）、高（≥30%） | Criteria of c-Myc Expression in Different Literatures | 1: Low expression (0-10%); 2: Moderate expression (10-30%); 3: High expression (≥ 30%) |
| 阴性（0）、局部（5-40%）、广泛（≥40%） |  | 1: Negative (0); 2: Focal (5-40%); 3: Extensive (≥ 40%) |
| 四分法（0-5%，5%-20%，20%-40%，≥40%） |  | 1：Negative: 0-20%;2：Positive: ≥ 20% |
| 0-20%，≥20% |  | 1：Negative: 0-20%;2：Positive: ≥ 20% |
| 0-30%，≥30% |  | 1：Negative: 0-30%;2：Positive: ≥ 30% |
| 0-40%，≥40% |  | 1：Negative: 0-40%;2：Positive: ≥ 40% |
| 5%；5-10%；10-30%；≥30% |  | 1: Negative (5%); 2: Low Expression (5-10%); 3: Moderate Expression (10-30%); 4: High Expression (≥ 30%) |
